# Supplementary material for: Carbon isotope budget indicates biological disequilibrium dominated ocean carbon storage at the Last Glacial Maximum
Source: Nat Commun. 2024 Sep 13;15:8006. doi: 10.1038/s41467-024-52360-z (PMC11393407; doi:10.1038/s41467-024-52360-z)
Supplement: Supplementary file 1 — Source Data [file 41467_2024_52360_MOESM1_ESM.zip › SourceData/SuppMat_NatComm.pdf]

# Supplementary information for "Carbon isotope budget indicates biological disequilibrium dominated ocean carbon storage at the LGM"

Anne Willem Omta, Christopher Follett, Jonathan Lauderdale, Raffaele Ferrari

August 14, 2024

The calculation of the relative contributions of the regenerated and disequilibrium carbon to the LGM-to-Holocene change in total biologically sequestered carbon uses the preformed $\delta^{13}$C of different water masses. To estimate the preformed $\delta^{13}$C of Northern-sourced water for the LGM and Holocene ($\bar{\delta}^{Holo}_{o,pre,N}$ and $\bar{\delta}^{LGM}_{o,pre,N}$), we use published benthic foraminifera $\delta^{13}$C from North Atlantic cores located at depths $< 2000$ m. We use benthic foraminifera $\delta^{13}$C from Southern Ocean cores located at depths $< 2000$ m to estimate the preformed $\delta^{13}$C of Southern-sourced water for the LGM and Holocene ($\bar{\delta}^{Holo}_{o,pre,S}$ and $\bar{\delta}^{LGM}_{o,pre,S}$). These data compilations (including citations) are provided in the Supplementary Material (SourceData.xlsx), along with the Matlab code with which Fig. 3 was generated (NatCommCode.zip).
