# Supplementary material for: Carbon isotope budget indicates biological disequilibrium dominated ocean carbon storage at the Last Glacial Maximum
Source: Nat Commun. 2024 Sep 13;15:8006. doi: 10.1038/s41467-024-52360-z (PMC11393407; doi:10.1038/s41467-024-52360-z)
Supplement: Supplementary file 3 — Description of Additional Supplementary Files [file 41467_2024_52360_MOESM3_ESM.pdf]

Description of Additional Supplementary Information

File Name: Supplementary Code 1

Description: The file 'CalcRegC_d13C_new.m' contains the Matlab code with which Figure 3 was created. In Matlab, it can be executed using the command 'CalcRegC_d13C_new'. The output file 'Dal.txt' contains Delta alpha (horizontal axis in Fig. 3), the output file 'Dra.txt' contains the center value of Delta m_{reg}/Delta m_{seq} (vertical axis in Fig. 3, dark blue line), the output file 'Dra_err.txt' contains the error estimate of Delta m_{reg}/Delta m_{seq} (vertical axis in Fig. 3, light blue shaded area). Please contact A.W. Omta (anne.omta@case.edu) for any questions.
